# Formation Mechanism of Legal Motivation Among College Students: A Moderated Mediation Model Involving Core Self-Evaluation and Social Support

**DOI:** 10.3390/bs15111548

**Published:** 2025-11-13

**Authors:** Shuhui Xu, Zhiqiang Wang

**Affiliations:** 1 Institute of Higher Education, Wenzhou University, Wenzhou 325035, China; 2 School of Teacher Education, Taizhou University, Taizhou 317000, China

**Keywords:** subjective social support, objective social support, legal motivation, core self-evaluation, college students, legal education

## Abstract

This research examines how perceived social support shapes the legal motivation of Chinese university students by unraveling the underlying psychological pathways. Integrating the relational legal socialization framework with self-determination theory, we test a moderated mediation model in which subjective social support, comprising emotional and informational resources from families, peers, and institutions, exerts both a direct effect on legal motivation and an indirect effect through core self-evaluation, which is characterized by stable, positive self-beliefs. Additionally, we investigate the role of objective social support, defined as concrete resources and formal assistance, in moderating the link between subjective support and core self-evaluation. Utilizing survey responses from 365 undergraduates across mainland China, mediation and moderated mediation analyses with bootstrapped confidence intervals demonstrate that subjective support significantly enhances legal motivation, partially via improvements in core self-evaluation. Crucially, the mediating influence of core self-evaluation is stronger when objective support is high, confirming the moderation hypothesis. These findings enrich legal socialization theory by bridging individual psychological processes with behavioral outcomes in a Chinese context and underscore the importance of simultaneously cultivating perceived support experiences and strengthening formal support structures to foster legal motivation and compliance among college students.

## 1. Introduction

Legal motivation refers to an individual’s intrinsic drive to understand, uphold, and apply the law, arising from the internalization of core legal values such as freedom, order, justice, and equality (Tyler, 2006; Xu & Yan, 2022), recent empirical research further indicates that this motivation can buffer the negative effects of moral disengagement on school bullying, functioning as a psychological protective factor that promotes law-abiding behavior (He et al., 2025). As Aristotle observed, “law is not a constraint but a tool for liberation and salvation,” underscoring how legal frameworks can transform abstract values into concrete motivation by safeguarding individual freedoms (Aristotle, 1997).

From a civic socialization perspective, the procedural justice model posits that fair and transparent legal procedures enhance perceptions of legitimacy, thereby nurturing intrinsic motivation for voluntary compliance rather than forced obedience (Trinkner et al., 2018; Tyler, 2006). In Tyler’s terms, legitimacy is “a quality of a rule or authority that leads others to feel a sense of obligation to voluntarily comply” (Tyler, 2003, p. 307), highlighting legal motivation as an internalized civic duty.

Understanding the formation of legal motivation is critical for effective citizenship education and the prevention of rule-violating behaviors. Gottfredson and Hirschi’s General Theory of Crime links deficits in self-control to deviant actions (Gottfredson & Hirschi, 1990). However, this study focuses on core self-evaluation, a stable appraisal of one’s own abilities and worth that is operationalized as an internal versus external locus of control, instead of self-control resources. Individuals high in core self-evaluation demonstrate a stronger internal locus of control, attributing successes and failures to their own capacities, which in turn promotes the internalization of legal norms (Judge et al., 2003).

Recent empirical work has begun to examine how social support and legal education influence compliance via attributional and motivational processes. Subjective social support, referring to the emotional and informational assistance perceived from family, peers, and institutions, can strengthen individuals’ internal locus of control and thus promote voluntary adherence to legal norms (Dağ & Şen, 2018; Khuan et al., 2024; Krause, 1987). Objective social support, which involves tangible resources and formal institutional assistance, may moderate the extent to which subjective support is transformed into stable self-beliefs, thereby defining the contextual conditions that enable or constrain the formation of legal motivation (Krause, 1997).

Accordingly, this study investigates how perceived social support shapes legal motivation among Chinese undergraduates by testing a moderated mediation model in which subjective social support influences legal motivation both directly and indirectly through core self-evaluation, which reflects the individual’s internal versus external locus of control, and in which objective social support moderates the pathway from subjective support to core self-evaluation. By clarifying the joint roles of perceived and structural supports in the development of legal motivation, the present research extends legal socialization theory and offers empirical guidance for legal education initiatives aimed at fostering an internal locus of control and sustained law-abiding behavior.

## 2. Theoretical Framework

### 2.1. Relationship Between Subjective Social Support and Legal Motivation

Subjective social support refers to the emotional and informational assistance individuals perceive receiving from family members, peers, and institutions, which can buffer negative affect during stress and adversity and thereby promote psychological well-being (Brissette et al., 2002; Zimet et al., 1988). Empirical evidence demonstrates that higher levels of perceived social support enable individuals to cope more effectively with stress, experience less anxiety, bolster self-esteem, and enhance self-efficacy, all of which yield positive mental-health outcomes (Malecki & Demaray, 2002; Orehek & Lakey, 2011; Sodeify & Moghaddam Tabrizi, 2020). For instance, students who report strong social support exhibit lower distress and adapt more readily to challenging circumstances than those with weaker support networks (Coplan et al., 2013; Xing et al., 2016). According to the social-buffering hypothesis, the mere perception of available interpersonal resources attenuates physiological and emotional stress responses (Cohen & Wills, 1985).

Within the legal-socialization process, subjective social support likewise plays a pivotal role. Authority-relationship theory posits that interactions with legal and non-legal authorities shape individuals’ legal attitudes and behaviors (Mladineo, 2019). Perceived social support reflects such interpersonal interactions, enhancing recognition of legal authority and thereby stimulating legal motivation. Moreover, the procedural-justice framework suggests that when individuals experience support in a procedurally fair legal environment, they are more inclined to identify with legal institutions and comply with rules (Fagan & Tyler, 2005; Trinkner & Cohn, 2014). Based on the theoretical framework outlined above, we hypothesize the following:

**Hypothesis** **1** **(H1):** *Students reporting higher subjective social support will exhibit greater legal motivation than those reporting lower support.*

### 2.2. Mediating Role of Core Self-Evaluation

Core self-evaluation (CSE) encompasses individuals’ fundamental appraisals of their self-esteem, self-efficacy, locus of control, and emotional stability (Judge & Bono, 2001). Higher CSE is associated with increased confidence and life satisfaction, as well as resilience in the face of stress (Li & Nie, 2010). Research indicates that social support can enhance self-efficacy and self-esteem, thus indirectly benefiting mental health (Zang et al., 2018), and that parental support satisfies basic psychological needs, fostering positive CSE development (Peng et al., 2021). Self-determination theory maintains that fulfillment of autonomy, competence, and relatedness needs drives adolescents toward self-enhancement, whereas thwarted needs precipitate self-undermining tendencies (Deci & Ryan, 2000). Based on the above theoretical framework, we further propose:

**Hypothesis** **2** **(H2):** *Higher subjective social support will predict higher core self-evaluation via satisfaction of basic psychological needs.*

High core self-evaluation, characterized by strong self-esteem, self-efficacy, internal control beliefs, and low neuroticism, has been linked to greater self-control (Bu et al., 2024; Guo, 2023). Conversely, low self-control correlates with diminished perceptions of legal legitimacy (Reisig et al., 2011). Because perceived legitimacy of legal authorities fosters voluntary legal compliance (Tyler, 2009) and deficits in self-control predict unlawful behavior (Reisig & Pratt, 2011; Reisig et al., 2011), we posit:

**Hypothesis** **3** **(H3):** *Higher core self-evaluation will be positively associated with legal motivation.*

Integrating H1–H3, we further propose that high subjective social support enhances CSE, which in turn increases legal motivation.

**Hypothesis** **4** **(H4):** *Core self-evaluation mediates the relationship between subjective social support and legal motivation.*

### 2.3. Moderating Role of Objective Social Support

Objective social support comprises tangible, observable resources such as material aid, network participation, and problem-solving guidance that individuals receive (Song & Fan, 2013; Xiao, 1994). Studies show subjective and objective support exert distinct influences on well-being (Jordan & Graham, 2012; X. Liu et al., 2007; Parrenas, 2001). Drawing on Conservation of Resources theory, which emphasizes resource acquisition and preservation as key motivators for growth (Hobfoll, 1989; Hobfoll et al., 2018), we argue that the availability of concrete support can strengthen individuals’ CSE. Considering the broader contextual factors that may influence these relationships, we propose the following:

**Hypothesis** **5** **(H5):** *Objective social support will moderate the effect of subjective social support on core self-evaluation, such that this effect is stronger under high (vs. low) objective support.*

Finally, we anticipate that increased objective support will enhance the indirect pathway from subjective support to legal motivation via CSE. Based on this anticipated moderated mediation, we further hypothesize:

**Hypothesis** **6** **(H6):** *Objective social support will positively moderate the mediating role of core self-evaluation in the link between subjective social support and legal motivation.*

### 2.4. The Current Study

**Figure 1:**
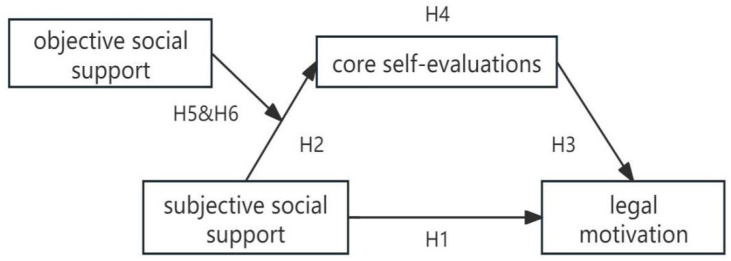
Illustrates the proposed moderated mediation model.

Based on the theoretical framework and the six proposed hypotheses, this study examines how perceived social support influences legal motivation in Chinese university students. We test a moderated mediation model (Figure 1). Subjective social support is expected to have direct effects on legal motivation and indirect effects through core self-evaluation. Objective social support is hypothesized to moderate the relationship between subjective support and core self-evaluation, as well as the indirect pathway to legal motivation. Survey data were collected from 365 undergraduates across mainland China. This study clarifies both direct and conditional indirect effects. It contributes to legal socialization theory and provides insights for fostering law-abiding behavior through perceived and formal support.

## 3. Method

### 3.1. Participants and Procedure

Undergraduate students enrolled in teacher-education programs at four universities in Zhejiang Province, China, completed a paper-and-pencil survey in spring 2024. After excluding incomplete or invalid responses, 365 valid questionnaires remained (149 freshmen, 169 sophomores, 43 juniors, 4 seniors; 105 male, 260 female). Data collection occurred during class under the supervision of trained assessors, who followed a standardized protocol emphasizing anonymity, confidentiality, and voluntary participation; each session lasted 15–20 min. All participants provided written informed consent. This study adhered to the principles of the Declaration of Helsinki and received approval from the Experimental Ethics Committee of the School of Education, Wenzhou University (Ref: WZU-2024-061).

### 3.2. Measures

Legal Motivation. The 22-item College Students’ Legal Motivation Scale (Xu & Yan, 2022) assesses three facets, learning, compliance, and application, on a 5-point Likert scale (1 = strongly disagree to 5 = strongly agree). The scale demonstrated excellent internal consistency in the present sample, with a total αof 0.986 and subscale α coefficients ranging from 0.873 to 0.929. Example items include “I strive to become a law-abiding citizen through adherence to legal statutes.” and “It is my belief that the application of law produces a deterrent effect.”

Core Self-Evaluation. The 10-item Core Self-Evaluation Scale (Du et al., 2012; Judge et al., 2003) uses a 5-point Likert format, with six items reverse-scored. A sample item is “I frequently feel down.” Higher total scores reflect more positive self-appraisals (α = 0.756).

Social Support. Subjective and objective support were measured with the Adolescent Social Support Scale (Ye & Dai, 2008), consisting of five items for perceived support and six for tangible aid, rated from 1 (not true) to 5 (true). A representative item is “I can often receive care and support from my classmates and friends.” Reliability was high for the overall scale (α = 0.901) and its subscales (αs = 0.893, 0.904).

The specific items for all scales are provided in the Supplementary Materials.

### 3.3. Data Analysis

All analyses were conducted in SPSS version 23.0, and mediation and moderation tests were performed using Hayes’ PROCESS macro (Model 4 and Model 7) (Hayes, 2013). Prior to hypothesis testing, the Shapiro–Wilk test was used to assess the normality of all continuous variables. Results indicated significant departures from normality for all measures: legal motivation (W = 0.948, *p* < 0.001), core self-evaluation (W = 0.918, *p* < 0.001), subjective social support (W = 0.941, *p* < 0.001), and objective social support (W = 0.900, *p* < 0.001). Accordingly, Spearman’s rank-order correlations were computed to examine pairwise associations among the study variables, including the demographic covariates gender and year.

The mediation hypothesis was tested using bias-corrected bootstrap confidence intervals based on 5000 resamples. Moderation effects were examined via interaction terms within the conditional process framework. Statistical significance for all inferential tests was set at *p* < 0.05.

## 4. Results

### 4.1. Assessment of Common Method Variance

To evaluate potential common method bias, we performed Harman’s one-factor test on all survey items. Exploratory factor analysis yielded seven factors with eigenvalues exceeding 1, collectively explaining 64.09% of the total variance. The first factor accounted for 31%—well below the 40% criterion—indicating that common method variance is unlikely to have substantially influenced the findings.

### 4.2. Tests for Demographic Variable Differences

To examine the influence of demographic variables on the primary study measures, we conducted Mann–Whitney U tests for gender differences and Kruskal–Wallis tests for grade-level differences. The Mann–Whitney U test indicated a significant gender difference only for core self-evaluation (W = 0.000267, *p* < 0.001, r = 0.191). No significant gender differences were observed for legal motivation, subjective social support, or objective social support (all *p* > 0.05). The Kruskal–Wallis tests revealed significant grade-level differences for legal motivation (H = 0.000244, *p* < 0.001, η^2^ = 0.045), subjective social support (H = 0.000542, *p* < 0.001, η^2^ = 0.040), and objective social support (H = 0.024, *p* < 0.05, η^2^ = 0.018). No significant grade-level differences were found for core self-evaluation (*p* = 0.221). Based on these results, grade level was included as a covariate in subsequent analyses.

### 4.3. Intercorrelations Among Key Variables

Spearman’s rank-order correlation analysis (Table 1) revealed that legal motivation was positively associated with core self-evaluation, subjective social support, and objective social support. Core self-evaluation likewise correlated positively with both forms of social support. Gender showed a positive relationship with core self-evaluation, whereas class year was negatively linked to legal motivation and both support variables. In light of prior evidence that males report lower perceived support than females (Grey et al., 2020), we controlled for gender and grade level in subsequent analyses.

### 4.4. Mediation Analysis of Subjective Social Support → CSE → Legal Motivation

Using Hayes’s PROCESS Model 4 (with gender and grade as covariates), subjective social support significantly predicted core self-evaluation (path a) and directly predicted legal motivation (path c). Even after accounting for CSE as a mediator, the direct effect of subjective support on legal motivation remained significant (path c′). Bootstrap estimates (5000 samples) produced 95% bias-corrected confidence intervals for both the direct effect and the indirect effect via CSE that excluded zero (see Table 2 and Table 3), confirming that CSE partially mediates the influence of subjective support on legal motivation.

### 4.5. Moderated Mediation with Objective Social Support

**Figure 2:**
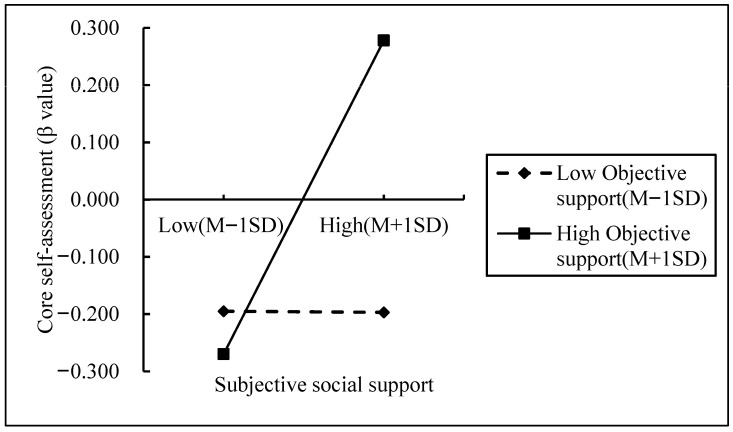
The Moderating Effect of Objective Social Support on the Relationship Between Subjective Social Support and Core Self-Evaluation. Note: Low Obiective support = Low Obiective social support, High Obiective support = High Obiective social support.

We then tested Model 7 to examine whether objective social support moderates the first stage (subjective support → CSE) of the mediation pathway. Controlling for gender and grade, the interaction between subjective and objective social support was a significant predictor of CSE (Table 4 and Table 5). Simple slope analyses (Figure 2) demonstrated that subjective social support had a non-significant effect on CSE under low objective support but a significant positive effect under high objective support. Thus, the strength of the indirect pathway from subjective support to legal motivation via CSE increases as objective social support rises.

## 5. Discussion

### 5.1. The Influence of Subjective Social Support on College Students’ Legal Motivation

The results showed that subjective social support significantly and positively predicted college students’ legal motivation, supporting Hypothesis 1. Prior research has shown that perceived social support can increase hope, a positive motivational state that sustains goal-directed behavior (Yao & Guo, 2018). Studies of migrant children also indicate that teacher and peer support predict integrity, defined here as a stable disposition toward honesty, trustworthiness and adherence to moral norms (J. Liu & Wang, 2018). Because integrity and legal motivation both reflect an inclination toward norm abiding behavior, these findings together suggest that perceived social support promotes legal motivation by strengthening prosocial values and motivational resources.

Social learning theory emphasizes that individuals learn and develop their behaviors through observing and imitating others (Annie, 2011; Bandura, 1977). Subjective social support typically originates from close relationships and significant others within an individual’s social network. The attitudes and behaviors of these significant others serve as powerful models for individuals. Through emotional attachment and the modeling effect of significant others, subjective social support makes individuals more likely to identify with legal and social norms, thereby strengthening their legal motivation. As a result, the higher an individual’s perceived subjective social support, the more likely they are to exhibit a strong motivation to learn, comply with, and apply the law (Bandura, 1977; Hirschi, 1996).

In addition to interpersonal modeling, the broader criminological and prevention literature indicates that policies addressing basic needs and strengthening legal awareness at the societal level can reduce offending and foster prolegal attitudes. Recent reviews and empirical studies have argued that promoting the fulfilment of basic human needs and enhancing legal consciousness are central components of effective crime prevention strategies (Abhishek & Balamurugan, 2024; Yanovska, 2023). Region-specific research further shows that legal awareness, understood as the internalization of legal norms and knowledge of rights and duties, plays a key role in preventing radicalization and promoting compliance with the law among young people (Cheung & Jia, 2024; Tymoshenko, 2025).

### 5.2. The Mediating Function of Core Self-Assessment

This investigation explored the intermediary role of core self-assessment in linking perceived social support with legal compliance motivation. The analysis revealed that core self-assessment served as a partial mediator in this association. Specifically, perceived social support exhibited both direct effects on legal motivation and indirect effects through the pathway of core self-assessment. These outcomes provide empirical validation for Hypotheses 2 through 4.

Core self-assessment represents a fundamental psychological resource encompassing four dimensions: self-worth perception, control orientation, emotional stability, and generalized competence belief (Judge & Bono, 2001). Research demonstrates that individuals scoring high on this composite trait typically display enhanced self-regard and an increased sense of life mastery (Chang et al., 2012; Wei et al., 2022). Those with elevated self-worth perception show greater alignment with societal standards and legal frameworks, as they associate law-abiding conduct with personal integrity and social validation. Similarly, individuals with strong generalized competence beliefs demonstrate greater initiative in lawful behaviors, including utilizing legal channels for conflict resolution, due to their confidence in achieving favorable results through these means.

Furthermore, people with an internal control orientation generally exhibit heightened ethical awareness, considering legal adherence both a personal responsibility and moral imperative. This perspective significantly strengthens their motivation for lawful conduct. Complementing this view, control theory posits that behavioral patterns are shaped by social connections, with robust support networks facilitating the internalization of normative standards (Hirschi, 1996). Consequently, enhanced perception of social support correlates with improved core self-assessment, which subsequently promotes greater legal compliance inclination.

Because emotional stability is one component of CSE, and because affective processes shape moral judgment and motivated behavior, we argue that emotion-regulation constructs are likely integral to the mediation pathway rather than peripheral correlates. Empirical work linking emotion-regulation difficulties to deontological moral judgments and to altered decision-making suggests that individuals with poorer regulation may convert social support into CSE less effectively or may apply CSE differently in legal contexts (Zhang et al., 2017; Szekely et al., 2015). Therefore, future models should explicitly test serial mediation (subjective support → emotion-regulation stabili → CSE → legal motivation) or moderated mediation (emotion-regulation difficulties moderating subjective support → CSE), which aligns this work with established findings on emotions and moral cognition (Dahò, 2025; Rullo et al., 2021; Zhang et al., 2017).

### 5.3. The Moderating Role of Objective Social Support

This study found that objective social support moderated the first stage of the mediation model. Specifically, when objective social support was low, subjective social support did not significantly predict core self-evaluation. However, when objective social support was high, the predictive effect of subjective social support on core self-evaluation became significant. That is, as individuals’ levels of objective social support increased, the predictive effect of subjective social support on core self-evaluation became more pronounced. This finding supports Hypotheses 5 and 6.

Social capital theory suggests that resources within social networks and relationships (social capital) significantly influence individual behavior. Under low objective social support, a lack of structural capital limits the effectiveness of cognitive capital (subjective support), as individuals lack the necessary resource network. In contrast, under high objective social support, sufficient structural capital allows cognitive capital (subjective support) to function more effectively, significantly enhancing core self-evaluation (Lin, 2001). Maslow proposed that human needs follow a hierarchical order, progressing from physiological needs to safety needs, social needs, esteem needs, and finally self-actualization needs. When lower-level needs are not met, higher-level needs may not function effectively (Maslow, 1943). Under conditions of low objective social support, basic needs (such as physiological and safety needs) may remain unfulfilled, limiting the effectiveness of subjective support (such as emotional support). Only when objective social support is high and basic needs are met can subjective support effectively influence higher-level needs (such as self-actualization), thereby significantly enhancing core self-evaluation. Therefore, for subjective social support to enhance legal motivation through core self-evaluation, it is essential to consider the level of objective social support. Only when a certain level of objective social support is available can subjective social support effectively predict core self-evaluation and subsequently exert a stronger influence on legal motivation.

This interactional finding dovetails with applied recommendations from criminology and legal education: policy and educational interventions that combine provision of tangible resources (e.g., community legal clinics, school-based legal education programs) with efforts to raise legal awareness and civic competence are likely to be most effective in cultivating law-abiding attitudes (Cheung & Jia, 2024). Likewise, systematic reviews linking childhood trauma and parental bonding to antisocial traits underscore that enhancing structural supports and early family interventions can alter developmental trajectories that otherwise predispose to low CSE and weak legal motivation (Schorr et al., 2020).

### 5.4. Theoretical and Practical Implications

Theoretically, this study refines understanding of legal motivation as an outcome of the dynamic interaction between relational and structural supports and internal psychological resources. By integrating the relational legal-socialization framework with self-determination theory, it demonstrates that subjective social support enhances legal motivation both directly and indirectly through core self-evaluation, and that this process is strengthened when objective support is high. These findings extend prior models of social capital and agency (Lin, 2001) and contextualize self-determination processes within the Chinese sociocultural environment, highlighting how perceived and structural supports jointly foster internalized legal adherence (Sheldon & Krieger, 2004).

Practically speaking, the results suggest that promoting legal motivation requires multilevel interventions that combine relational and structural strategies such as enhancing emotional and informational support through mentorship and peer networks while ensuring access to concrete institutional resources such as legal counselling and rights workshops. Such integrated approaches can convert perceived support into stable self-beliefs and law-abiding behavior, particularly when emotion regulation and personality factors are taken into account (Rullo et al., 2021; Zhang et al., 2017). At the policy level, strengthening legal awareness and meeting basic needs can further consolidate these effects and assist in the prevention of delinquency (Abhishek & Balamurugan, 2024; Tymoshenko, 2025), and evaluation designs that incorporate behavioral indicators and test mediation–moderation pathways will enhance both theoretical precision and practical impact (Cheung & Jia, 2024; Preacher et al., 2007).

### 5.5. Limitations and Strengths

Limitations. The cross-sectional design limits causal inference and temporal sequencing; future research should employ longitudinal or experimental designs to establish directionality (Maxwell & Cole, 2007). The exclusively Chinese undergraduate sample restricts cross-cultural generalizability, as cultural norms such as Confucian authority orientation may moderate the observed relations (Sheldon & Krieger, 2004). Reliance on self-reports introduces potential common-method and social-desirability biases; subsequent studies should incorporate behavioral indicators and multi-informant assessments to enhance validity. Moreover, key theoretically relevant variables—such as emotion regulation (DERS), moral identity, religiosity, and personality traits like conscientiousness—were not measured, though these may act as critical moderators or mediators (Rullo et al., 2021; Szekely et al., 2015; Zhang et al., 2017). Finally, heterogeneity in early-life adversity, which relates to antisocial traits, suggests that baseline vulnerability may shape responsiveness to support; future longitudinal work should examine the moderating role of childhood trauma and parental bonding (Schorr et al., 2020).

Strengths. This study advances the field by integrating subjective and objective social support within a unified conditional process framework, thereby moving beyond simple correlational models. The finding that objective support amplifies the impact of subjective support on core self-evaluation provides a clear entry point for policy and educational interventions. The use of validated scales enhances construct validity and facilitates cross-study comparison. Theoretically, by linking social support processes with legal motivation, the study bridges psychological theory and civic-education practice, offering empirically grounded directions for future longitudinal and intervention research (Rullo et al., 2021; Zhang et al., 2017).

## 6. Conclusions and Future Directions

### 6.1. Conclusions

By unpacking the mechanisms through which perceived social support fosters legal motivation—specifically via enhancement of internal locus of control and under the amplifying effect of tangible, structured assistance—this study offers a nuanced model of support-driven civic engagement. Emotional and informational backing from family, peers, and institutions not only directly motivates students to learn, obey, and apply legal norms, but also operates indirectly by strengthening their sense of personal agency. The presence of objective resources, such as legal clinics and institutional guidance, magnifies these effects, underscoring the value of integrated support frameworks in legal education. Overall, the findings suggest that reducing delinquency and promoting law-abiding citizenship requires coordinated interventions that combine micro-level psychological supports with macro-level resource provision and legal-awareness initiatives.

### 6.2. Future Directions

To build cumulative theory and generate policy-relevant evidence, future work should prioritize designs that establish temporal precedence, test mechanisms involving affective capacity, and evaluate multilevel interventions. First, longitudinal panel studies with at least three waves (e.g., baseline, 6 months, 12 months) across culturally diverse samples are needed to test a longitudinal moderated-mediation model in which perceived social support predicts later core self-evaluations (CSE), which in turn predict subsequent legal motivation; emotion-regulation difficulties (e.g., DERS) are hypothesized to attenuate the conversion of subjective support into CSE (i.e., DERS moderates the subjective support → CSE path), while conscientiousness and religiosity are expected to explain incremental variance in legal motivation beyond CSE (Dahò, 2025; Rullo et al., 2021; Zhang et al., 2017). Analytically, researchers should use cross-lagged SEM with bootstrapped indirect effects and latent interactions, and assess measurement invariance across sites to ensure cross-cultural comparability (Little, 2013).

Second, to test causality and intervention utility, randomized controlled trials should evaluate combined, multilevel interventions that pair structural facilitation (e.g., legal clinics, institutional guidance) and mentorship with brief emotion-regulation training, compared against support-only and control conditions; the expectation is that combined interventions will produce larger increases in CSE and legal motivation, with ΔCSE mediating program effects and baseline emotion-regulation difficulties moderating the mediated pathway (Cheung & Jia, 2024; Preacher et al., 2010). Outcomes should include both self-report (CSE, legal motivation) and behavioral indicators (engagement with legal resources) and be examined with multilevel mixed-effects models and multilevel mediation techniques.

Third, developmental and adversity perspectives should be integrated: research with adolescents and community youth should examine whether childhood trauma profiles and patterns of parental bonding predict lower baseline CSE and reduced responsiveness to supports and interventions; such moderators can identify groups needing early-family or trauma-informed components to restore developmental trajectories (Schorr et al., 2020; Yanovska, 2023).

Collectively, these approaches will clarify whether emotion-regulation is a boundary condition or a mechanistic mediator in the social-support → CSE → legal-motivation process, and will test whether combining structural, relational, and intrapersonal strategies yields durable increases in law-abiding orientations across contexts (Gangemi et al., 2025; Rullo et al., 2021; Zhang et al., 2017).

## Figures and Tables

**Table 1:** Descriptive Statistics and Correlation Analysis of Variables.

Variables	1	2	3	4	5	6
1. Gender	1					
2. Grade Level	−0.100 *	1				
3. Legal motivation	−0.224 ***	0.042	1			
4. Core self-evaluations	−0.017	0.191 ***	0.163 **	1		
5. Subjective social supports	−0.105 *	−0.031	0.431 ***	0.183 ***	1	
6. Objective social supports	−0.141 **	−0.043	0.399 ***	0.140 **	0.689 ***	1
M ± SD			92.21 ± 12.58	35.02 ± 6.76	19.46 ± 3.26	24.73 ± 3.69

Note: * *p* < 0.05, ** *p* < 0.01, *** *p* < 0.001.

**Table 2:** Mediation Model Test of Core Self-Evaluation.

Variables	Legal Motivation		Core Self-Evaluations		Legal Motivation	
	*β*	*t*	*β*	*t*	*β*	*t*
constant	0.319	1.166	0.585	1.9582	0.387	1.413
Subjective social supports	0.404	8.488 ***	0.185	3.590 ***	0.426	9.037 ***
Core self-evaluations	0.116	2.427 *				
Gender	0.009	0.086	−0.421	−3.602 ***	−0.040	−0.371 **
Grade Level	−0.208	−3.117 **	0.047	0.648	−0.202	−3.015
*R* ^2^	0.229		0.067		0.216	
*F*	21.334 ***		6.505 ***		24.858 ***	

Note: *** *p* < 0.001, ** *p* < 0.01, * *p* < 0.05.

**Table 3:** Decomposition of Total Effect, Direct Effect, and Mediation Effect.

	Effect	BootSE	BootLLCI	BootULCI
Total effect	0.426	0.047	0.333	0.519
Direct effect	0.404	0.048	0.311	0.498
Indirect effect	0.022	0.013	0.002	0.052

**Table 4:** Moderated Mediation Model Test.

Variables	Legal Motivation		Core Self-Evaluations	
	*β*	*t*	*β*	*t*
constant	0.319	1.166	0.415	1.40
Gender	0.009	0.086	−0.379	−3.264 **
Grade Level	−0.208	−3.117 **	0.052	0.721
Subjective social supports	0.404	8.488 ***	0.137	1.944
Core self-evaluations	0.116	2.427 *		
Objective social supports			0.027	1.397
Subjective supports × Objective supports			0.037	3.720 ***
*R* ^2^	0.229		0.104	
*F*	21.334 ***		6.913 ***	

Note: *** *p* < 0.001, ** *p* < 0.01, * *p* < 0.05.

**Table 5:** Mediation Effects at Different Levels of Objective Social Support.

	Indicator	Effect	BootSE	BootLLCI	BootULCI
Moderated mediation effects	eff1 (M − 1SD)	0.000	0.048	−0.106	0.092
	eff2 (M)	0.061	0.047	−0.010	0.170
	eff3 (M + 1SD)	0.123	0.070	0.012	0.281
Comparison of the Effects	eff2-eff1	0.062	0.037	0.006	0.148
	eff3-eff2	0.124	0.075	0.013	0.296
	eff3-eff2	0.062	0.037	0.006	0.148

## Data Availability

The data that support the findings of this study are available from the corresponding author upon reasonable request.

## Supplementary Materials

The following supporting information can be downloaded at: https://www.mdpi.com/article/10.3390/bs15111548/s1.
