# Supplementary material for: Formation Mechanism of Legal Motivation Among College Students: A Moderated Mediation Model Involving Core Self-Evaluation and Social Support
Source: Behav Sci (Basel). 2025 Nov 13;15(11):1548. doi: 10.3390/bs15111548 (PMC12649237; doi:10.3390/bs15111548)
Supplement: Supplementary file 1 [file behavsci-15-01548-s001.zip › behavsci-3834605-supplementary.pdf]

The Chinese Version of the Scales

同学，你好！

非常感谢你参加大学生法律动机问卷调查，此问卷旨在了解目前大学生法律动机的现状，答案无对错之分，所以请同学们依据您最真实的情况和意见来填写。同时我们承诺对你的作答进行保密，且你的回答只会用于科学研究，请放心作答。

题目	完全不符合	比较不符合	不确定	比较符合	完全符合
Q1.我觉得法治是一种令人感到满意的治国理政的方式。	1	2	3	4	5
Q2.我觉得，使用法律使社会更有秩序。	1	2	3	4	5
Q3.我学习法律是为了培育法治思维。	1	2	3	4	5
Q4.我认为培育“法律至上”的观念很必要。	1	2	3	4	5
Q5.我认为守法是因为法律具有公正性。	1	2	3	4	5
Q6.我认为养成“任何人不得享有法律之外的特权”的观念很必要。	1	2	3	4	5
Q7.课后我经常关注与法律相关的知识。	1	2	3	4	5
Q8.我常想，如果不遵守法律，就容易出现不好的后果。	1	2	3	4	5
Q9.我学习法律的主要动力是想使我国成为一个法治社会。	1	2	3	4	5
Q10.我遵守法律是觉得这是现代公民应有的素质。	1	2	3	4	5
Q11.通过学习，我懂的法律知识比一般同学要多。	1	2	3	4	5
Q12.我想通过遵纪守法成为一名合格的公民。	1	2	3	4	5
Q13.我常因懂得更多的法律知识而产生强烈的满足感。	1	2	3	4	5
Q14.我因努力学习法律知识而感到踏实。	1	2	3	4	5
Q15.我觉得，法律的使用可以抚平伤痛。	1	2	3	4	5
Q16.我觉得遵守法律是令人愉快的事情。	1	2	3	4	5
Q17.我觉得，法律使用可以具有震慑效果。	1	2	3	4	5
Q18.我觉得，很多人认为遵纪守法利大于弊。	1	2	3	4	5
Q19.对法律的了解，我对法律的学习兴趣越来越浓了。	1	2	3	4	5
Q20.我觉得，使用法律使个人生活更美好。	1	2	3	4	5
Q21.我经常通过看法律专业书籍而有意识地提高自己的法律素养。	1	2	3	4	5
Q22.我觉得，使用法律是社会进步的表现。	1	2	3	4	5

核心自我评价量表，本部分用于考察个体对自我价值与能力的总体评价与感受。

题目	非常不同意				非常同意
Q1.我相信自己在生活中能获得成功。	1	2	3	4	5
Q2.我经常感觉到情绪低落。	1	2	3	4	5
Q3.失败时，我感觉自己很没用。	1	2	3	4	5
Q4.我能成功地完成各项任务。	1	2	3	4	5
Q5.我觉得自己对工作（学习）没有把握。	1	2	3	4	5
Q6.总的来说，我对自己满意。	1	2	3	4	5
Q7.我怀疑自己的能力。	1	2	3	4	5
Q8.我觉得自己对事业上的成功没有把握。	1	2	3	4	5
Q9.我有能力处理自己的大多数问题。	1	2	3	4	5
Q10.很多事情我都觉得很糟糕、没有希望。	1	2	3	4	5

社会支持量表，本部分用于测量学生从家庭、朋友及他人处感受到的社会支持程度。

题目	非常不符合				非常符合
Q1.大多数同学都很关心我。	1	2	3	4	5
Q2.面对两难的选择时，我会主动向他人寻求帮助。	1	2	3	4	5
Q3.当有烦恼时，我会主动向家人、亲友倾诉。	1	2	3	4	5
Q4.我经常能得到同学、朋友的照顾和支持。	1	2	3	4	5
Q5.当遇到困难时，我经常会向家人、亲人寻求帮助。	1	2	3	4	5
Q6.我周围有许多关系密切、可以给予我支持和帮助的人。	1	2	3	4	5
Q7.在我遇到问题时，同学、朋友会出现在我身边。	1	2	3	4	5
Q8.在困难的时候，我可以依靠家人或亲友。	1	2	3	4	5
Q9.我经常从同学、朋友那里获得情感上的帮助和支持。	1	2	3	4	5
Q10.我经常能得到家人、亲友的照顾和支持。	1	2	3	4	5
Q11.需要时，我可以从家人、亲友那里得到经济支持。	1	2	3	4	5
Q12.当遇到麻烦时，我通常会主动寻求别人的帮助。	1	2	3	4	5
Q13.当我生病时，总能得到家人、亲友的照顾。	1	2	3	4	5
Q14.当有烦恼时，我会主动向同学、朋友倾诉。					
Q15.在我遇到问题时，家人、亲友会出现在我身旁。	1	2	3	4	5
Q16.我经常从家人、亲友那里获得情感上的帮助和支持。	1	2	3	4	5
Q17.当遇到困难时，我经常会向同学、朋友寻求帮助	1	2	3	4	5

The English Version of the Scales

Dear student,

Thank you very much for participating in the College Students' Legal Motivation Questionnaire. The purpose of this questionnaire is to understand the current status of legal motivation among college students. There are no right or wrong answers, so please respond according to your true situation and opinions. We assure you that your responses will be kept strictly confidential and used solely for scientific research. Please feel free to answer with confidence.

Scale: 1 = Strongly disagree; 2 = Disagree; 3 = Neither agree nor disagree; 4 = Agree; 5 = Strongly agree.

Q1. I believe the rule of law is a satisfactory approach to governing.
Q2. I believe that applying the law makes society more orderly.
Q3. I study law to cultivate a rule-of-law mindset.
Q4. I consider it necessary to promote the concept of the supremacy of law.
Q5. I obey the law because I believe the law is just.
Q6. I consider it important to cultivate the idea that no one should enjoy privileges beyond the law.
Q7. I often follow legal-related information after class.
Q8. I often think that failing to comply with the law is likely to lead to negative consequences.
Q9. My primary motivation for studying law is to contribute to making my country a society governed by the rule of law.
Q10. I comply with the law because I consider it a quality that modern citizens should possess.
Q11. Through my studies, I have greater legal knowledge than the average student.
Q12. I want to become a competent citizen by abiding by laws and regulations.
Q13. I often feel strong satisfaction from knowing more about the law.
Q14. I feel secure when I make efforts to learn legal knowledge.
Q15. I believe that the application of law can help remedy harms.
Q16. I find complying with the law to be a pleasant experience.
Q17. I believe that the application of law can have a deterrent effect.
Q18. I think many people believe that adhering to the law brings more benefits than harms.
Q19. The more I learn about the law, the more interested I become in studying it.
Q20. I believe that applying the law makes personal life better.
Q21. I regularly read professional legal texts to consciously improve my legal competence.
Q22. I believe that the application of law is a sign of social progress.

Core Self-Evaluation Scale，This section examines individuals' fundamental self-assessments of their self-worth and capabilities.

Instruction: Please indicate the extent to which each statement describes you.
Scale: 1 = Strongly disagree; 2 = Disagree; 3 = Neutral; 4 = Agree; 5 = Strongly agree.

Item	Statement
Q1	I believe I can achieve success in life.
Q2	I often feel down or depressed.
Q3	When I fail, I feel that I am useless.
Q4	I am capable of successfully completing various tasks.
Q5	I feel uncertain about my ability to handle work or study tasks.
Q6	Overall, I am satisfied with myself.
Q7	I often doubt my own abilities.
Q8	I feel uncertain about achieving success in my career.
Q9	I am able to handle most of my own problems.
Q10	I often feel that many things are terrible or hopeless

Perceived Social Support Scale，This section evaluates the degree of social and emotional support that students perceive from their family, friends, and significant others.

Instruction: Please indicate how well each statement describes your real situation.
Scale: 1 = Strongly disagree; 2 = Disagree; 3 = Neutral; 4 = Agree; 5 = Strongly agree.

Item	Statement
Q1	Most of my classmates care about me.
Q2	When facing a difficult choice, I actively seek help from others.
Q3	When I am troubled, I take the initiative to talk with my family or close friends.
Q4	I often receive care and support from classmates and friends.
Q5	When facing difficulties, I often seek help from my family or relatives.
Q6	There are many people around me with whom I have close relationships and who can offer me support and help.
Q7	When I encounter problems, my classmates and friends are there for me.
Q8	In times of difficulty, I can rely on my family or close friends.
Q9	I often receive emotional support and help from my classmates and friends.

Item	Statement
Q10	I often receive care and support from my family and relatives.
Q11	When needed, I can obtain financial support from my family or relatives.
Q12	When I encounter trouble, I usually seek help from others.
Q13	When I am ill, I always receive care from my family or close friends.
Q14	When I am troubled, I take the initiative to talk with my classmates or friends.
Q15	When I encounter problems, my family or close friends are there for me.
Q16	I often receive emotional support and help from my family or close friends.
Q17	When I face difficulties, I often seek help from my classmates or friends.
